# Methylprednisolone, venous thromboembolism, and association with heparin to 30 days in hospital survival in severe Covid-19 pneumonia

**DOI:** 10.1186/s12890-021-01810-1

**Published:** 2022-01-06

**Authors:** Ronaldo C. Go, Themba Nyirenda, Maryam Bojarian, Davood K. Hosseini, Mehek Rahim, Kevin Kim, Keith M. Rose

**Affiliations:** 1 Hackensack Meridian School of Medicine, Nutley, NJ USA; 2 Hackensack Meridian School of Medicine and Department of Critical Care, Hackensack University Medical Center, Hackensack, NJ USA; 3 Office of Research Administration, Hackensack Meridian Health, Hackensack, NJ USA; 4 Department of Internal Medicine, Hackensack University Medical Center, Hackensack, NJ USA

**Keywords:** COVID-19, Methylprednisolone, Venous thromboembolism, Heparin

## Abstract

**Background:**

Mortality in severe COVID-19 pneumonia is associated with thrombo-inflammation. Corticosteroids are given to attenuate the inflammation, but they are associated with thrombosis. The aims of this study were to determine the risk of venous thromboembolism between no methylprednisolone and methylprednisolone (dose versus duration) and to evaluate any synergistic dose-dependent association of heparin and methylprednisolone to 30 days in hospital survival.

**Methods:**

This was a secondary analysis of a retrospective cohort. Patients included in this study were ≥ 18 years of age and admitted for severe COVID-19 pneumonia between March and June 2020 in 13 hospitals in New Jersey, United States. A propensity score analysis between administration of methylprednisolone and no methylprednisolone was fitted for 11 variables and Youden Index Method was used to determine cut-off between low dose and high dose methylprednisolone. Multivariate cox regression was to assess risk.

**Results:**

In 759 patients, the incidence of venous thromboembolism was 9% of patients who received methylprednisolone and 3% of patients who did not receive methylprednisolone with a [RR 2.92 (95% CI 1.54, 5.55 P < 0.0001)]. There was a higher incidence of mechanical ventilation in the methylprednisolone group. The median d-dimer between patients with venous thromboembolism was higher compared to those without (P < 0.0003). However, the d-dimer was not statistically significant between those who had venous thromboembolism between methylprednisolone and no methylprednisolone groups (P = 0.40). There was no higher risk in high dose versus low dose [RR = 0.524 (95% CI 0.26, 1.06 P 0.4)]; however, the risk for venous thromboembolism between methylprednisolone for > 7 days and ≤ 7 days was statistically significant (RR 5.46 95% CI 2.87, 10.34 P < 0.0001). Patients who received low dose methylprednisolone and therapeutic heparin had a trend towards higher risk of mortality compared to prophylactic heparin (HR 1.81 95% CI 0.994 to 3.294) (P = 0.0522). There was no difference in 30 days in hospital survival between high dose methylprednisolone with prophylactic or therapeutic heparin (HR 0.827 95% CI 0.514 to 1.33) (P = 0.4335).

**Conclusion:**

Methylprednisolone for > 7 days had a higher association of venous thromboembolism. There was no added benefit of therapeutic heparin to methylprednisolone on mechanically ventilated patients.

**Supplementary Information:**

The online version contains supplementary material available at 10.1186/s12890-021-01810-1.

## Introduction

As of July 2, 2021, there has been 182,319,261 coronavirus disease 2019 (COVID-19) cases and 3,954,324 deaths worldwide [1]. It is a biphasic disease, with predominant viral shedding during the first week and hyperinflammation during the second week. The main cause of mortality is acute respiratory distress syndrome (ARDS). Lung histologic studies show severe endothelial damage, diffuse alveolar damage, thrombosis in situ, intussusceptive angiogenesis, and patterns of organizing pneumonia (OP) and / or acute fibrosing organizing pneumonia (AFOP) [2, 3]. There is a higher degree of thrombosis compared to ARDS secondary to influenza [2, 3].

Corticosteroids were considered for their anti-inflammatory and anti-fibrotic effect and due to the Recovery study, has become standard of care [4]. During the initial pandemic surge there were concerns with corticosteroids prolonging viral shedding and were used at our institution as “rescue” therapy or reserved for patients who were hypoxic or requiring higher amounts of oxygen support and are at higher risk of dying. It was at the discretion of the ordering provider at what dose and at what oxygen support. There has been no consensus on the definition of low dose and high dose methylprednisolone. Low dose methylprednisolone has been defined as 40 mg IV daily for 5 days [5] and 80 mg IV daily for 8 days [6, 7]. High dose methylprednisolone has been defined as 120–180 mg IV daily for 10 days [8], 250 mg IV every day for 3 days [9], 250–500 mg IV daily for 3 days followed by prednisone 50 for 10 days [10], and 1 mg/kg IV for ≥ 3 days [11].

In our initial study on real world data, there were heterogeneity in methylprednisolone administration. We used Youden’s index to determine the cut off between low dose (LDMP) and high dose (HDMP). We found that LDMP (< 1.36 mg/kg of absolute body weight/day), administered > 7 days from onset of symptoms was associated with prolonged survival compared to no methylprednisolone and no additional benefit with HDMP (≥ 1.36 mg/kg of absolute body weight/day) [12]. Interestingly, the studies with varying definitions of high dose and showed mortality benefit fit our actual weight-based definition of low dose [8–11].

Non-COVID-19 studies have suggested a corticosteroid associated coagulopathy due to high levels of Factor VIII, Factor IX, and von Willebrand factor (vWF) and enhanced thrombin generation [13–16]. Some studies suggest that higher doses of methylprednisolone were associated with higher rates of thrombosis [17–19]. Two studies suggest that risk of venous thromboembolism (VTE) decreases with duration of use [20, 21]. A recent meta-analysis in COVID-19 suggested an association of VTE with corticosteroids, although the association may also be due to severity of underlying disease [22]. There was significant heterogeneity in corticosteroid administration, and they were not able to assess which regimen was associated with higher risk [22].

The incidence of VTE in COVID-19 ranges 1.31–17% [23–25]. Higher risk of thrombotic complications may suggest benefit from higher doses of anticoagulation. Heparin maybe an ideal anti-coagulation because of its anti-thrombotic, anti-inflammatory, and anti-viral properties. One observational study showed that intermediate to high prophylactic dose reduced the incidence of thrombotic complications [26]. Another study showed that intermediate to higher prophylactic doses reduced thrombotic complications but were not associated with improved mortality [27]. Early therapeutic anticoagulation in critically ill patients have failed to show improved survival [28, 29]. Therapeutic anticoagulation was shown to improve survival in patients who are not critically ill or on low flow nasal cannula [30].

Since mortality in COVID-19 ARDS is associated with thrombo-inflammation, there may be a synergistic, dose dependent association with prolonged in hospital survival with methylprednisolone and heparin. Currently, there is sparsity in literature regarding this outcome.

## Methodology

### Eligibility criteria

We performed a comprehensive review of real-world data collected within Hackensack Meridian Health (HMH), a NJ health network comprising of thirteen hospitals. Data was obtained from electronic health records (EHR) in patients with COVID-19. We included only adult patients, hospitalized for at least 2 days between March 1, 2020, and June 15, 2020, with a positive SARS- CoV-2 PCR and diagnosed with severe pneumonia, defined as SpO_2_ < 94% on room air at sea level, a respiratory rate > 30 breaths/min, PaO_2_/FiO_2_ < 300 mm Hg, or lung infiltrates > 50%. We excluded pregnant patients. Approval was obtained by the Hackensack Meridian Health Institutional Review Board (study #Pro2020-0485) and the study was also registered on ClinicalTrials.Gov as a prospective observational database (NCT04347993).

### Data collection process and data items

Age, gender, race, ethnicity, and sex were self-reported. Weight and height were measured. Comorbidities were defined prior to COVID-19 and included cardiovascular disease, lung disease, diabetes mellitus, neurologic disease, cancer, and renal disease. SARS-CoV-2 was detected by nasal PCR. Routine blood work included complete blood count (CBC), complete metabolic profile (CMP), magnesium, phosphate, troponin, arterial blood gases, Beta natriuretic peptide, C-Reactive Protein (CRP), D-dimer, ferritin, and interleukin 6. Demographics, clinical characteristics, laboratory values, treatments, and outcomes were manually abstracted. Data was entered and maintained using REDCap (Research Electronic Data Capture) hosted by HMH. Data abstraction occurred daily from June 1, 2020, to December 1, 2020.

### Outcomes

The primary outcomes were the risk of thromboembolic disease between no methylprednisolone and methylprednisolone (dose versus duration) and the association of methylprednisolone with or without therapeutic dose of heparin and 30-days in hospital survival.

### Data analysis

A one-to-one propensity score matched design between those treated with no methylprednisolone and those treated with methylprednisolone was constructed [12]. Patients were matched based on variables associated with mortality such as age groups (age ≥ 60 years vs. age < 60 years), obesity (BMI ≥ 30.0 kg/m^2^ vs. BMI < 30.0 kg/m^2^), sex (Male/Female), diabetes (Yes/No), hypertension (Yes/No), cancer (Yes/No), respiratory rate (respiratory rate > 22 vs < 22), renal failure (Yes/No), oxygen saturation (< 94% vs.  ≥ 94%), CRP (> 20 mg/dL vs ≤ 20 mg/dL), and quick sequential organ failure assessment or qSOFA (score: 0,1,2,3) [12]. A nearest-neighbor (greedy match) used a caliper of 0.20 to obtain the matched sample. We performed a post-match assessment of how distribution of propensity scores (or logit of propensity scores) and the adjusted variables are balanced between the no methylprednisolone and methylprednisolone using standardized difference and variance ratio and graphical displays produced by the ASSESS statement of PROC PSMATCH in SAS 9.4 [12]. Youden index was used to determine the cut off between LDMP and HDMP.

Categorical variables were presented as frequency and percentage. Continuous variables were presented as median and interquartile range (IQR). Shapiro–Wilk test was used to assess normality of continuous variables. Time to events such as in-hospital survival, start of mechanical ventilation, and discharge were obtained using Kaplan–Meier method which reported median (95% confidence interval), 30 days survival rates (95% CI), and the intervals were calculated using the arcsine-square root transformation method. Comparison of the propensity matched samples was performed using stratified log-rank test based on quintiles of the propensity scores as the strata. To examine association of risk factor of interest, methylprednisolone treatment, Cox proportional hazard regression analysis with robust covariance [31] (sandwich estimator) to account paired observations was conducted and hazard ratios (95% CIs), and p values were reported in all univariable and multivariable analysis from PROC PHREG. The proportional hazard (PH) assumption, critical in Cox regression, was evaluated using a Kolmogorov-type supremum test [31] in ASSESS statement of PROC PHREG. If the PH assumption was violated, then a continuous variable which also violated the PH and its interaction with time were included in the model to adjust for the significant interaction with time to the risk of in-hospital mortality [32].

## Results

Between March 4 and June 15, 2020, 2041 patients were flagged in the electronic health record with a diagnosis of COVID-19 and pneumonia. A total of 539 patients were excluded based on eligibility criteria (< 18 years of age, pregnant, received other formulations of corticosteroids, or hospitalized for less than 2 days). Thus, 1121 patients had their data abstracted. In the unmatched population, the median duration of symptoms prior to admission was 5 days in the no methylprednisolone and methylprednisolone group (P < 0.0165) and there were more patients on invasive mechanical ventilation in methylprednisolone group (P > 0.0001).

A propensity score matched sample was constructed out of 759 patients (380 in no methylprednisolone and 379 in methylprednisolone) [12] (Table 1 and Additional file 1: Table S2 and Fig. S1). An examination of the proportional hazard assumption, methylprednisolone, and Fractional inspired oxygen (FiO2) significantly violated it (both with P < 0.0001). Data on P/F ratio was lacking; and FiO2 was used since 95% of patients had this data [12]. FiO_2_ is a time dependent covariate that can account also for the level of oxygen support. The supremum test also indicated that non- proportionality was observed in other variables such as nursing home, lack of taste or smell, WBC < 11,000 cells/ml, creatinine > 1.5 ng/mL, respiratory rate > 22 bpm, hydroxychloroquine (HCQ), methylprednisolone, HDMP, LDMP, calcium and initial diastolic blood pressure. All variables with non-proportional hazard were adjusted using FiO2 in both no methylprednisolone and methylprednisolone groups.

**Table 1:** Characteristics of hospitalized COVID-19 patients treated with or without Methylprednisolone (n = 759)

Level	No methylprednisolone (n = 380)	Methylprednisolone (n = 379)	P value
	Count (%)	Count (%)	
Age in years	65 (54, 80)	64 (55, 74)	0.0230
Age ≥ 60 years	254 (66.67)	244 (64.55)	0.2436
Male	237 (62.20)	242 (64.02)	0.5775
Weight (kg)	81.67 (70.30, 96.00)	83.90 (71.70, 99.80)	0.8758
BMI ≥ 30 kg/m^2^	179 (46.98)	181 (47.88)	0.6767
Black	58 (15.30)	44 (11.80)	0.2500
White	178 (46.97)	160 (42.90)	0.2500
Asian/Indian	25 (6.60)	133 (35.66)	0.2500
Hispanic	118 (31.13)	181 (47.88)	0.2500
Non-smoker	278 (78.31)	251 (74.04)	0.2334
Smoker (former or current)	77 (21.69)	88 (25.96)	0.2334
Fever	250 (65.79)	294 (77.78)	0.0003
Shortness of breath	248 (65.09)	298 (79.05)	< .0001
Cough	242 (63.68)	270 (71.43)	0.0191
Altered mental status	63 (16.54)	41 (10.85)	0.0032
GI	76 (20.00)	81 (21.49)	0.5211
Anosmia or ageusia	6 (1.59)	9 (2.45)	0.5930
Duration of symptoms PTA in days	5.00 (2.00, 7.00)	5.00 (3.00, 7.00)	0.0699
Diabetes	143 (37.53)	138 (36.51)	0.6521
COPD	20 (5.25)	28 (7.41)	0.2482
Asthma	24 (6.30)	37 (9.81)	0.0741
Hypertension	225 (59.06)	219 (57.94)	0.5525
Cancer	43 (11.29)	43 (11.38)	0.9013
Cerebrovascular accident	18 (4.74)	14 (3.70)	0.3692
Coronary artery disease	29 (7.61)	28 (7.41)	0.8886
Arrhythmia	41 (10.79)	30 (7.94)	0.1213
Renal failure	28 (7.35)	31 (8.20)	0.6682
Rheumatologic disorder	10 (2.62)	19 (5.04)	0.0588
qSOFA 0	224 (58.49)	222 (58.42)	0.7647
qSOFA 1	130 (33.94)	130 (34.21)	0.7647
qSOFA 2	28 (7.31)	26 (6.84)	0.7647
qSOFA 3	1 (0.26)	2 (0.53)	0.7647
Temperature	98.80 (97.70, 100.40)	99.30 (98.00, 100.70)	0.1284
Heart rate	95.00 (84.00, 108.00)	97.00 (86.00, 108.00)	0.1438
Arterial pressure	92.33 (83.33, 100.50)	90.67 (81.83, 99.33)	0.0870
Respiratory rate	19.00 (18.00, 22.00)	20.00 (18.00, 22.00)	0.3231
O2 Sat < 94%	215 (56.43)	217 (57.41)	0.6733
Nasal cannula	160 (82.05)	131 (65.83)	0.2500
High flow	6 (3.08)	15 (7.54)	0.2500
CPAP	1 (0.51)	2 (1.01)	0.2500
BiPAP	0 (0.00)	2 (1.01)	0.2500
Non-rebreather	26 (13.33)	46 (23.12)	0.2500
Mechanical ventilation	35 (11.15)	129 (39.33)	< .0001
WBC	6.65 (5.10, 9.20)	6.50 (5.10, 9.50)	1.0000
HGB	13.40 (12.30, 14.50)	13.50 (12.20, 14.70)	0.6746
PLT	203.00 (161.00, 257.00)	189.00 (147.00, 252.00)	0.2736
ALC	0.90 (0.60, 1.30)	0.80 (0.60, 1.10)	0.0031
IL6	12.00 (5.00, 39.00)	15.00 (5.00, 36.00)	0.2678
CRP	9.88 (4.99, 17.31)	12.67 (6.84, 19.08)	0.0047
D-dimer	1.09 (0.65, 2.20)	1.44 (0.72, 3.13)	0.1155
Ferritin	729.50 (325.50, 1404.00)	867.00 (418.00, 1548.00)	0.0675
Creatinine	1.01 (0.80, 1.50)	1.01 (0.80, 1.35)	0.2630
Troponin	0.03 (0.01, 0.30)	0.02 (0.01, 0.09)	0.0732
BNP	131.85 (40.30, 1000.55)	88.80 (26.20, 362.00)	0.2110
Hydroxychloroquine	269 (71.93)	317 (88.55)	< .0001
Azithromycin	255 (68.55)	264 (73.54)	0.0728
Remdesivir	3 (0.82)	10 (2.82)	0.0196
Tocilizumab	13 (3.53)	63 (17.65)	< .0001
Convalescent plasma	0 (0.00)	4 (28.57)	< .0001

Out of 754 patients with available anticoagulation data, 392 (52%) received prophylactic heparin (Pac) and 232 (30.8%) received heparin drip or therapeutic enoxaparin (Tac). 130 (17.2%) patients did not receive any anticoagulation therapy. 36 patients (27.27%) who received no anticoagulation, 88 patients (22.39%) who received prophylactic heparin, and 117 (50%) who received therapeutic heparin expired (P < 0.0001). Therapeutic anticoagulation was mostly initiated due to clinical suspicion or confirmation of VTE [worsening hypoxia or echocardiographic findings of right heart strain and/or radiographic evidence of deep venous thrombosis (DVT) or pulmonary embolism (PE)], atrial fibrillation, or acute coronary syndrome. Therefore, there was a higher proportion of patients on oxygen support who also received therapeutic heparin.

9% of patients who received methylprednisolone [LDMP (N = 25) and HDMP (N = 10)] and 3% of patients who did not receive methylprednisolone (N = 12), had VTE [RR 2.92 (95% CI 1.54, 5.55 P < 0.0001)]. The relative risk between HDMP vs LDMP [RR 0.524 (95% CI 0.26, 1.06 P = 0.4)] was not statistically significant (Tables 2, 3). The relative risk for VTE between methylprednisolone for > 7 days (N = 24) and ≤ 7 days (N = 11) was significant [RR 5.46 (95% CI 2.87, 10.34 P < 0.0001)]. The median d-dimer in patients without VTE (0.9775 mcq/mL) and patients with VTE (4.707 mcq/mL) was statistically significant (P < 0.0003). However, the median d-dimer in the methylprednisolone group with VTE (7.115 mcq/mL) and no methylprednisolone with VTE (5.37 mcg/mL) was not statistically significant (P = 0.40).

**Table 2:** Differences in rates of DVT/PE across the Methylprednisolone Administration

Outcome	NMP	MP	P value
DVT	9 (2.33)	34 (9.07)	
PE	3 (0.78)	1 (0.27)	< .0001
None	375 (96.90)	340 (90.67)

**Table 3:** Differences in rates of DVT/PE across the Methylprednisolone Dose Levels

Outcome	NMP	LD MP	HD MP	P value
DVT	9 (2.33)	25 (11.74)	9 (5.56)	
PE	3 (0.78)	0 (0.0)	1 (0.62)	< .0001
None	375 (96.90)	188 (88.26)	152 (93.83)	

**Fig. 1:**
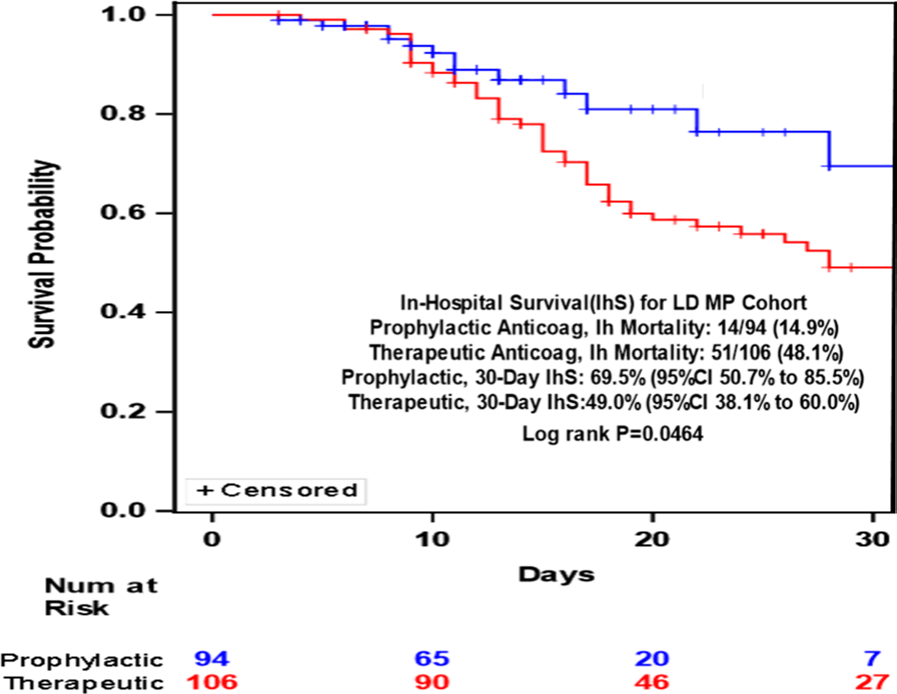
Kaplan–Meier plot of LDMP with prophylactic heparin versus therapeutic heparin and 30 days in hospital survival

**Fig. 2:**
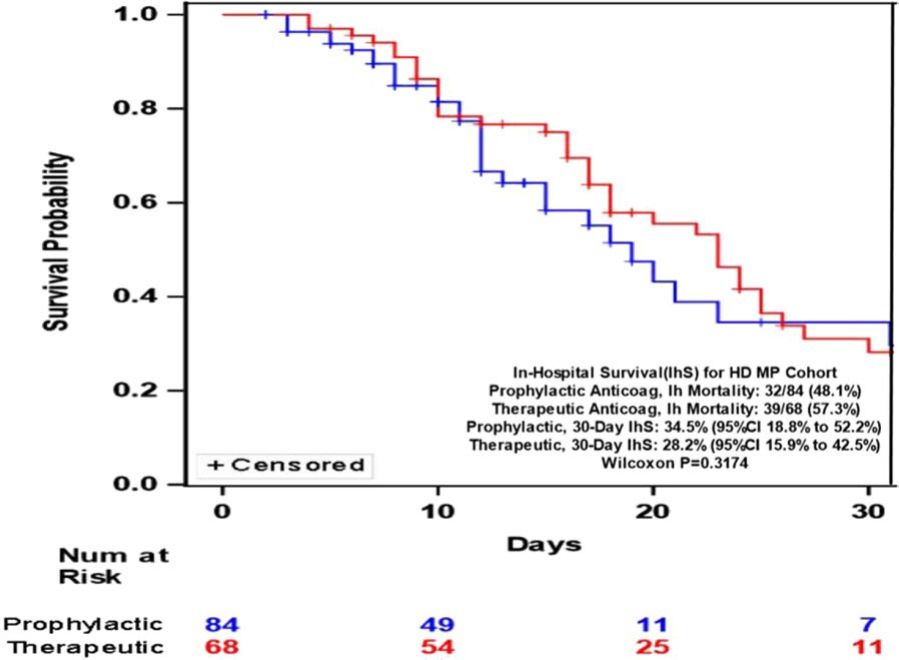
Kaplan–Meier Plot of HDMP with prophylactic heparin versus therapeutic heparin and 30 days in hospital survival

In patients who received prophylactic heparin only, 14 patients (16.09%) with no oxygen support, 11 patients (10.19%) with non-invasive oxygen support, and 17 patients (89.47%) with invasive oxygen support or mechanical ventilation expired (P < 0.0001). In patients who received methylprednisolone and prophylactic heparin, 10 patients (16.67%) with no oxygen support, 6 patients (8.70%) with non-invasive oxygen support, and 30 patients (61.22%) with invasive oxygen support expired (P < 0.0001). In patients who received methylprednisolone and therapeutic heparin, 6 patients (25%) with no oxygen support, 9 patients (45%) with non-invasive oxygen support, and 11 patients (78.57%) with invasive oxygen support or mechanical ventilation expired (P = 0.0059) (Additional file 1: Tables S3–S6). Patients who received LD MP and therapeutic heparin had a trend towards higher risk of in hospital mortality (HR 1.81 95% CI 0.994–3.294) (P = 0.0522) compared to LDMP and prophylactic heparin (Fig. 1). There was no difference in 30 days in hospital survival between HDMP with prophylactic or therapeutic heparin (HR 0.827 95% CI 0.514 to 1.33) (P = 0.4335) (Fig. 2). There were 5 patients who had major bleeding episodes. Heparin was stopped but the main cause of mortality for all patients was ARDS.

## Discussion

There was a higher risk of venous thromboembolism in patients who received methylprednisolone compared to no methylprednisolone use. This may be due to higher production of procoagulant factors from corticosteroids. Cushing’s syndrome, which is due to excess levels of endogenous glucocorticoids, is associated with elevated levels of procoagulant Factor VIII, Factor IX and von Willebrand Factor [33–35]. In vitro studies of exogenous corticosteroids revealed increased synthesis and secretion of VWF and plasminogen-activator-1 (PAI-1). One study suggested corticosteroids had elevated FVII, FVIII, and FXI while cumulative evidence suggested lowered levels of VWF and fibrinogen and increased levels of PAI-1 [35]. It is interesting to note that the pulmonary pathologic studies that showed higher levels of thrombosis with COVID-19 compared to Influenza had patients that were treated with corticosteroids [2, 3].

Another possible explanation is that the association of methylprednisolone with VTE maybe due to the severity of underlying inflammatory disease. Severity may be suggested with higher median CRP and higher oxygen support in the methylprednisolone group compared to no methylprednisolone group. There was a higher frequency of corticosteroids in those requiring invasive mechanical ventilation. This was like the finding from another study from that time, although they used dexamethasone [36]. Regardless of methylprednisolone use or not, patients with VTE had higher median d-dimers compared to those with no VTE. This suggest that elevated d-dimer is independently associated with VTE [37–42]. D-dimer has also been used to assess severity of the disease [37–42].

One of the strengths of this study is that we were able to determine which methylprednisolone variable was associated with VTE. HD MP was not associated with higher risk of VTE compared to LD MP, but methylprednisolone > 7 days had a higher risk of VTE compared to ≤ 7 days. This association with VTE may diminish with increasing methylprednisolone use [20, 21]. At > 1 year duration, the initial procoagulant effects are overshadowed by the inhibition of platelet aggregation and tissue factor-mediated leukocyte procoagulant [20, 21, 43]. However, all our patients required < 30 days of corticosteroids.

Our study suggested therapeutic heparin was not superior to prophylactic heparin, regardless of the methylprednisolone dose to 30 days in hospital survival. Most of these patients required higher level of oxygen support. Our findings are like the conclusions of the two large, randomized trials in non-critically ill and critically ill patients [29, 30]. Both suggest that survival benefit for patients on low flow nasal cannula but not on patients requiring at least high flow nasal cannula [29, 30]. Heparin has multiple non-anticoagulant mechanisms that maybe beneficial in COVID-19 earlier in disease course. It is an analogue of haparan sulphate. This is a cofactor that is required by ACE-2 mediated entry of SARS-COV-2 into the cell. Therefore, heparin may block binding of the virus into the cell [44–46]. This anti-viral affect might be more beneficial if given during the first week of illness when viral shedding is at its peak. Heparan sulphate provides a negatively charge dependent function of the glycocalyx and is degraded by heparanase [47]. Heparanase is increased in COVID-19 and contributes to endothelial dysfunction with increased leakage. Heparin inhibits heparanase. Heparin also neutralizes cytokine and chemokine synthesis and function, complement activation, and histones which are responsible for apoptosis [47].

## Limitations

Our study has several limitations. First, since it is an observational study, we cannot draw causal inferences due to known and unknown confounders. However, propensity matching was employed to limit the known confounders. Second, misclassification of data is possible due to manual extraction of structured and unstructured data from medical health records. Third, there was also a higher prevalence of invasive mechanical ventilation, and benefits of therapeutic heparin with methylprednisolone for patients on nasal cannula could not be seen.

## Conclusion

Duration > 7 days of methylprednisolone is associated with higher risk of venous thromboembolism. This may be due to prolonged duration and severity of inflammation of the underlying disease. Regardless of whether the patient received methylprednisolone or not, higher levels of d-dimer were associated with venous thrombosis. There was no synergistic benefit of methylprednisolone and therapeutic heparin for severe COVID-19 requiring higher oxygen support. Therapeutic heparin should be reserved for patients with radiographic evidence of venous thrombo-embolic disease.

## Supplementary Information

**Additional file 1**. Supplemental Section with Figure S1 and Tables S1–S6.

## Data Availability

The datasets used and/or analyzed during the current study are available from the corresponding author on reasonable request.

## Abbreviations

AFOP: Acute fibrinous organizing pneumonia
ARDS: Acute respiratory distress syndrome
BMI: Body mass index
CBC: Complete blood count
CI: Confidence interval
CMP: Complete metabolic profile
Covid-19: Coronavirus disease 2019
CRP: C-reactive protein
EHR: Electronic Health Record
FiO2: Fraction of inspired oxygen
HCQ: Hydroxychloroquine
HMH: Hackensack Meridian Health
HR: Hazard ratio
IQR: Interquartile range
HDMP: High dose methylprednisolone
LDMP: Low dose methylprednisolone
OP: Organizing pneumonia
Pac: Prophylactic dose of heparin
PAI-1: Plasminogen activation factor 1
PaO2: Partial pressure of arterial oxygen
qSOFA: Quick sequential organ failure assessment
RR: Relative risk
SARS-COV-2: Severe acute respiratory syndrome coronavirus 2
SpO2: Oxygen saturation
Tac: Therapeutic dose of heparin
vWF: von Wildebrand factor
