# Supplementary material for: Methylprednisolone, venous thromboembolism, and association with heparin to 30 days in hospital survival in severe Covid-19 pneumonia
Source: BMC Pulm Med. 2022 Jan 6;22:6. doi: 10.1186/s12890-021-01810-1 (PMC8731184; doi:10.1186/s12890-021-01810-1)
Supplement: Supplementary file 1 — Additional file 1. Supplemental Section with Figure S1 and Tables S1–S6. [file 12890_2021_1810_MOESM1_ESM.pdf]

Supplemental Section for

Methylprednisolone, Venous Thromboembolism, and Association with Heparin to 30 days in Hospital Survival in Severe COVID-19 Pneumonia

Ronaldo C. Go, Themba Nyirenda, Maryam Bojarian, Davood Karimi Housseini, Mehek Rahim, Kevin Kim, and Keith M. Rose

Table of Contents

Figure S1. Cohort Flow Chart 3

Table S1. Baseline Demographics Disease Characteristics on Unmatched Population 3

Table S2. Level of Oxygen Support with Treatment……………………………………………...6

Table S3. Level of Oxygen Support and No methylprednisolone and Prophylactic anticoagulation ....

Table S4. Level of Oxygen Support for No Methylprednisolone and Therapeutic Anticoagulation……………………………………………………………………………….36

Table S5. Level of Oxygen Support and Methylprednisolone and Prophylactic Anticoagulation 36

Table S6. Level of Oxygen Support and Methylprednisolone and Therapeutic Anticoagulation 37

Figure S1. Cohort Flow Chart.

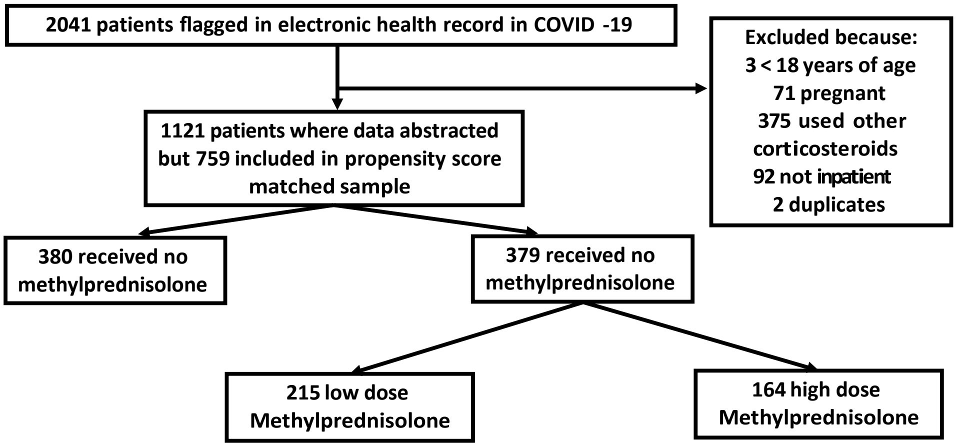

Table S1. Baseline Demographics Disease Characteristics on Unmatched Population

Variable	Unmatched No methylprednisolone (N=645)	Unmatched Methylprednisolone (N=476)	P Value
Age in years	64.00(53.00,79.00)	64.00(56.00,73.00)	0.6620
Male	411(63.43)	219(53.03)	0.6079
Weight (kg)	81.20(68.70,90.72)	83.90(71.10,99.80)	0.0040
BMI (kg/m^2)	28.18(24.83,31.65)	29.48(25.94,34.30)	<.0001
Nursing Home	125(19.38)	43(10.26)	<.0001
Former/Current smoker	116(19.86)	96(25.81)	0.0377
Never smoker	468(80.14)	276(74.19)	0.0377
SOB	412(63.78)	334(78.96)	<.0001
Cough	417(64.75)	301(71.16)	0.0327
AMS	109(17.33)	46(11.11)	0.0058
GI	149(23.17)	88(20.90)	0.4077
Anosmia or Ageusia	10(1.57)	9(2.20)	0.4834
Duration of Symptoms prior to admission	5.00(2.00,7.00)	5.00(3.00,7.00)	0.0165
Diabetes	214(33.18)	157(37.20)	0.1888
COPD	27(4.19)	30(7.09)	0.0506
Asthma	40(6.22)	40(9.48)	0.0569
Cancer	61(9.46)	49(11.61)	0.2594
CAD	86(13.33)	67(15.84)	0.2839
CVA	28(4.35)	15(3.55)	0.6336
CHF	51(7.96)	32(7.57)	0.9071
Arrhythmia	65(10.12)	38(8.96)	0.5967
Renal Failure	40(6.23)	33(7.78)	0.3249
Rheumatologic Disease	15(2.33)	22(5.20)	0.0159

qSOFA 0	375(59.62)	225(54.35)	0.2544
qSOFA 1	199(31.64)	156(37.68)	0.2544
qSOFA 2	51(8.11)	31(7.49)	0.2544
qSOFA 3	4(0.64)	2(0.48)	0.2544
O2 sat < 94%	305(48.41)	225(57.25)	0.0069
Temperature	99.00(98.00,100.70)	99.30(98.00,100.80)	0.0476
Heart Rate	95.00(82.00,108.00)	95.00(82.00,108.00)	0.0581
Respiratory Rate	19.00(18.00,21.00)	20.00(18.00,22.00)	0.0097
Nasal Cannula	227(82.85)	137(66.50)	0.0005
Venti mask	4(1.46)	3(1.46)	0.0005
High Flow	8(2.92)	15(7.28)	0.0005
CPAP	1(0.36)	2(0.97)	0.0005
BPAP	0(0.00)	2(0.97)	0.0005
Mechanical Ventilation	55(10.62)	138(39.88)	<.0001
WBC	6.50(5.00,9.10)	6.50(5.10,9.50)	0.5947
HGB	13.40(12.00,14.50)	13.50(12.20,14.80)	0.2190
PLT	200.00(158.00,251.00)	186.00(147.00,251.00)	0.0596
ALC	0.90(0.60,1.20)	0.79(0.60,1.10)	0.0007
IL6	11.50(5.00,34.00)	12.00(5.00,32.00)	0.6607
CRP	10.91(5.20,20.79)	13.11(7.09,20.20)	0.0444
D-Dimer	1.01(0.64,2.11)	0.98(0.61,1.89)	0.7909
Ferritin	641.89(320.65,1453.60	838.96(430.40,1569.80)	0.0044
Creatinine	1.00(0.80,1.40)	1.01(0.80,1.33)	0.9379
Troponin	0.03(0.01,0.30)	0.02(0.01,0.09)	0.0516
BNP	103.70(29.85,701.30)	88.80(26.20,362.00)	0.1702
Hydroxychloroquine	463(73.73)	333(88.33)	<.0001
Azithromycin	438(70.19)	277(73.47)	0.2793
Remdesivir	4(0.65)	63(16.80)	0.0061
Tocilizumab	31(5.01)	11(2.94)	<.0001
Convalescent Plasma	0(0.00)	4(28.57)	0.0002
ECMO	1(0.17)	9(2.43)	0.0011
Dialysis	19(3.09)	11(2.92)	0.8748

HR = Hazard Ratio; CI = Confidence Interval; SOB = Shortness of Breath; AMS = Altered Mental Status; GI = Gastrointestinal Symptoms; PTA = Prior to admission; COPD = Chronic Obstructive Disease; CAD = Coronary Artery Disease; CVA = Cerebrovascular Accident; CHF = Congestive Heart Failure; LFTs = elevated liver function tests; qSOFA = Quick Sepsis Related Organ Failure Assessment; HCQ = Hydroxychloroquine; AZ = Azithromycin; MP = Methylprednisolone; HD MP = High Dose Methylprednisolone; LD MP = Low Dose Methylprednisolone; WBC= White Blood Cells; HGB = Hemoglobin; PLT = Platelet; ALC = Absolute Lymphocyte Count; ECMO = Extracorporeal Membrane Oxygenation

Table S2. Level of Oxygen Support with Treatment (Methylprednisolone and Anticoagulation)

	MP + Anticoagulant (n=754)						
**Oxygen Support**	**Total (n=754)**	**NMP, NAc (n=106)**	**NMP, PAc (n=214)**	**NMP, TAc (n=58)**	**MP, NAc (n=24)**	**MP, PAc (n=178)**	**MP, TAc (n=174)**
**None**	256	44<br>41.51	87<br>40.65	24<br>41.38	12<br>50.00	60<br>33.71	29<br>16.67
**Non-Invasive Oxygen Support**	282	54<br>50.94	108<br>50.47	20<br>34.48	9<br>37.50	69<br>38.76	22<br>12.64
**Invasive Mechanical-Ventilation**	216	8<br>7.55	19<br>8.88	14<br>24.14	3<br>12.50	49<br>27.53	123<br>70.69

Abbreviations: NAc = No anticoagulation; Pac = Prophylactic anticoagulation;

Tac = Therapeutic anticoagulation

Table S3. Level of Oxygen Support and No methylprednisolone and Prophylactic anticoagulation.

Out of 214 hospitalized patients, 42(19.6%) in the NMP+ PAc arm. The difference in in-hospital mortality between the levels of supplemental oxygen: None (16.1%) vs Non-invasive oxygen support (10.2%) vs invasive mechanical ventilation (89.5%%), was significant (p<0.0001)

No methylprednisolone + Prophylactic Anticoagulant (n=214)				
	**Level of Oxygen Support**			
**Expired**	**None (n=87)**	**Non-invasive oxygen support (n=108)**	**Invasive Mechanical-Ventilation (n=19)**	**P-value**
**Alive**	73<br>83.91	97<br>89.81	2<br>10.53	<.0001
**Expired**	14<br>16.09	11<br>10.19	17<br>89.47	

Table S4. Level of Oxygen Support for No Methylprednisolone and Therapeutic Anticoagulation.

26 of 58 (44.8%) in the NMP+ Therapeutic Anticoagulant group. The difference in in-hospital mortality between the levels of supplemental oxygen: None (25.0%) vs non invasive oxygen support (45.0%) vs mechanical ventilation (78.6%) was significant(p=0.0059)

	No Methylprednisolone and Therapeutic Anticoagulation (N=58)			
	Level of Oxygen Support			
Expired	None (N=24)	Non Invasive Oxygen Support (N=20)	Invasive Mechanical-Ventilation (N=14)	P-value
Alive	18<br>75.00	11<br>55.00	3<br>21.43	0.0059
Expired	6<br>25.00	9<br>45.00	11<br>78.57	

Table S5. Level of Oxygen Support and Methylprednisolone and Prophylactic Anticoagulation.

46 of 178(25.8%) in the MP+ Prophylactic Anticoagulant group. The difference in in-hospital mortality between the levels of supplemental oxygen: None (16.7%) vs non invasive oxygen support(8.7%) vs invasive mechanical ventilation (61.2%) was significant(p<0.0001)

Methylprednisolone + Prophylactic Anticoagulant (n=178)				
	Supplemental Oxygen			
Expired	None (n=60)	Non-invasive oxygen support (n=69)	Invasive Mechanical Ventilation (n=49)	P-value
Alive	50<br>83.33	63<br>91.30	19<br>38.78	<.0001
Expired	10<br>16.67	6<br>8.70	30<br>61.22	

Table S6. Level of Oxygen Support and Methylprednisolone and Therapeutic Anticoagulation

90 of the 174 (51.7%) who were treated with MP + therapeutic anticoagulants expired during the COVID-19 hospitalization. The difference in in-hospital mortality between the levels of supplemental oxygen: None (16.7%) vs. Non-mechanical ventilation (8.7%) vs. mechanical ventilation (61.2%) was significant (P<0.0001)

Methylprednisolone + Therapeutic Anticoagulant(n=174)				
	Supplemental Oxygen			
Expired	None (n=29)	Non invasive oxygen support (n=22)	Invasive Mechanical-Ventilation (n=123)	P-value
Alive	22<br>75.86	18<br>81.82	44<br>35.77	<.0001
Expired	7<br>24.14	4<br>18.18	79<br>64.23	
